# Heterogeneity of Soft Tissue Sarcomas and Its Implications in Targeted Therapy

**DOI:** 10.3389/fonc.2020.564852

**Published:** 2020-09-23

**Authors:** Xin-Hui Du, Hua Wei, Peng Zhang, Wei-Tao Yao, Qi-Qing Cai

**Affiliations:** ^1^Department of Orthopedics, The Affiliated Cancer Hospital of Zhengzhou University, Henan Cancer Hospital, Zhengzhou, China; ^2^Department of Anesthesiology, Pain and Perioperative Medicine, The First Affiliated Hospital of Zhengzhou University, Zhengzhou, China

**Keywords:** soft tissue sarcoma, tumor heterogeneity, targeted therapy, clinical features, genomic alterations

## Abstract

Soft tissue sarcomas are a set of malignancies of mesenchymal origin. Due to the rarity and similarity in clinical presentation, they are grouped together and treated similarly in clinic. The response rates for current chemotherapy are around 20% and the median overall survival for advanced soft tissue sarcoma are less than 2 years. Thus, the current strategy with identical treatment for all soft tissue sarcomas is far from satisfactory. In this study, we first reviewed the current clinical and genomic findings of soft tissue sarcoma, paying special attention to the heterogeneities among different tumors. Then we reviewed the state-of-art understanding of targeted therapy in soft tissue sarcoma. We observed tremendous heterogeneity both in clinical and genomic settings between different tumors. Individualized treatment plans demonstrated better response and disease control and should be advocated. In summary, heterogeneity of soft tissue sarcomas requires the development of individualized treatment plans such as targeted therapy.

## Background

Soft tissue sarcomas are a collection of highly aggressive malignancies often present with slow growing lumps with or without pain (1). They can occur in all age groups and account for 1% of cancers in adults and 15% of cancers in children (2). Imaging and blood test results are mostly non-specific and can’t be used for differential diagnosis. The proper diagnosis largely depends on the features under microscope. Based on the immunohistochemistry markers and patterns of tumor cell arrangement, this group of rare diseases are divided into more than 50 different histological types (1). Due to the low incidence but complexity of this collection of diseases, the differential diagnosis can be very challenging. In fact, a large portion of soft tissue sarcomas are named undifferentiated pleomorphic sarcoma (UPS) because no defining features could be found.

Most of the soft tissue sarcomas are treated in the same way in clinic regardless of the pathological types. Surgery with or without radiotherapy is the first line treatment for localized disease (3). Non-resectable tumors may become resectable after induction therapy with necrosis factor alpha- and melphalan- based isolated limb perfusion technique (4). Radiotherapy can be used in a preoperative, intraoperative (5–7), or postoperative manner (8). The addition of radiotherapy has been shown to be able to reduce tumor volume and help local disease control (9, 10).

For advanced or metastatic disease, systematic treatment such as chemotherapy based on doxorubicin are recommended (11). However, the reported response rates are only about 20%. The prognosis of advanced soft tissue sarcomas is dismal, with the median overall survival of less than 2 years even with the doxorubicin treatment (12). Thus, it is urgent to find better cure for these diseases.

Despite some common features shared among soft tissue sarcomas, they are indeed different diseases and shouldn’t be arbitrarily grouped together and receive the same therapy. In fact, separating soft tissue sarcomas and treat different diseases individually have already shown to result in better disease control in some cancer types (13, 14).

In this review, we searched for sarcoma related research paper within the last 10 years in Pubmed and summarized recent updates on clinical and genomic findings of soft tissue sarcomas with the emphasis on heterogeneity among different diseases. Heterogeneity is a universal feature in cancer and we mainly focus on the differences between different sarcoma types in this paper. In addition, we also reviewed current understanding of targeted therapy in soft tissue sarcoma.

## Heterogeneity Among Different Sarcomas

### In Clinical Settings

As mentioned previously, soft tissue sarcomas consist of more than 50 different histological types, each with unique clinical manifestations and prognosis. Different types of sarcoma are inclined to affect patients of different age groups. For example, rhabdomyosarcomas are most commonly seen in children and epithelioid sarcoma are commonly seen in young adults. However, other sarcomas such as angiosarcoma, leiomyosarcoma, and liposarcoma mainly affect older people (2). Sarcoma heterogeneity is also shown as different patients or even different lesions in the same patient would present different responses to the same treatment, which is very common in the clinic.

Different sarcomas show different clinical courses in terms of metastasis. Sarcomas such as dermatofibrosarcoma protuberans can recur several times without metastasis (3). Some sarcoma types tend to metastasize to regional lymph nodes such as epithelioid sarcoma (15), while other sarcomas tend to metastasize early through the blood to lungs such as alveolar sarcoma of soft parts and synovial sarcoma (16).

### In Genomic Settings

The genomic alterations are also different among various sarcomas. Soft tissue sarcomas can be grossly divided into two broad genetic groups: those with simple karyotypes harboring specific genetic alterations and those with complex karyotypes. The first group of diseases often present with recurrent chromosome translocations resulting in fusion genes and protein. The fusion genes and proteins are mostly tumor-promoting and serve as diagnostic factors in the differential diagnosis in tumors such as synovial sarcoma (17). The second group of diseases often present with significant genomic instability and copy number alterations such as liposarcoma. Both gene copy number gain and loss are detected in different cancer types. For example, tumor suppressor genes such as TP53 and RB1 are found mutated while amplification of MDM2, CKD4 are detected in various sarcoma types (1).

Specific genomic alterations have been identified in certain tumor types. The loss of SMARCB1/INI1 protein in epithelioid sarcoma is tumor-promoting and is currently used in the clinic as a biomarker for differential diagnosis (18). More importantly, the loss of SMARCB1/INI1 leads to the deregulation of EZH2, and drugs targeting EZH2 have been proven successful in controlling tumor growth and are currently approved to be used in treating epithelioid sarcoma (19). Dedifferentiated liposarcoma is characterized by 12q13-15 amplifications. Synovial sarcoma is a simple karyotype defined by the translocation t(X,18) (p11, q11), which results in an SS18-SSX fusion protein that disrupts epigenetic regulation. Synovial sarcoma displayed very few somatic copy number alterations or mutations. But SS18-SSX1 or SS18-SSX2 fusion are identified in all synovial sarcoma cases. Other fusion transcripts such as TRIO-TERT are also detected in sarcomas (20).

## Targeted Therapy

As mentioned above, different sarcomas vary greatly in genomic alterations. Based on these alterations, multiple targeted therapies have been developed and approved such as pazopanib (21), anlotinib (22), and most recently tazemetostat (19). Pazopanib and anlotinib are both multi-target tyrosine kinase inhibitors. Pazopanib was mostly tested in advanced non-adipocytic soft tissue sarcomas and showed significantly prolonged progression-free survival (4.6 months vs. 1.6 months) comparing with the placebo control group (23). Anlotinib has been tested in clinical trials containing multiple advanced sarcoma types. Disease control was observed with a 12-week progression-free survival of 68% and an objective response rate of 13% (24).

Tazemetostat is the first-in-class, small molecule enhancer of zeste homolog 2 (EZH2) inhibitor approved in the United States in January 2020 to treat locally advanced or metastatic epithelioid sarcoma. The drug produced responses in 15% of patients in a phase II trial, and 67% of the responses lasted at least 6 months. It is approved for the treatment of adults and adolescents aged > = 16 years with unresected epithelioid sarcoma (25).

However, researches with targeted therapy were not without setbacks. Olaratumab is a human anti-platelet derived growth factor receptor alpha monoclonal antibody which has shown antitumor activity in human sarcoma xenograft models. Initially, the overall survival benefit was observed in a phase 2 clinical trial with soft tissue sarcomas (12). However, most recently a phase 3 trial compared the overall survival of 509 adults advanced soft tissue sarcomas treated with doxorubicin plus olaratumab or placebo. Median overall survival of total soft tissue sarcomas or leiomyosarcoma alone was not significantly different (Total STS 20.4 vs. 19.7 months, leiomyosarcoma 21.6 vs. 21.9 months). They concluded that patients with advanced soft tissue sarcoma have a median overall survival of fewer than 2 years and adding olaratumab to doxorubicin does not improve the overall survival (26).

## Conclusion

Soft tissue sarcomas show tremendous heterogeneity both in clinical and genomic settings and thus should be treated separately. Targeted therapy based on tumor types and genomic findings showed promising results.

## Author Contributions

X-HD and HW drafted the manuscript. PZ, W-TY, and Q-QC helped to revise the manuscript. All authors contributed to the article and approved the submitted version.

## Conflict of Interest

The authors declare that the research was conducted in the absence of any commercial or financial relationships that could be construed as a potential conflict of interest.
